# Supplementary material for: Nomenclature for Tracking of Genetic Variation of Seasonal Influenza Viruses
Source: Influenza Other Respir Viruses. 2026 Feb 13;20(2):e70230. doi: 10.1111/irv.70230 (PMC12904685; doi:10.1111/irv.70230)
Supplement: Supplementary file 1 — Figure S1: Tangle‐gram of recent H1N1pdm isolates focusing on subclade H1‐D of HA on the left with the NA tree on the right. Subclade nomenclatures for both segments allows for straightforward identification of multi‐segment constellations. Table S1: A(H3N2) HA clade definitions. Table S2: A(H1N1)pdm09 HA clade definitions. TABLE S3: B/Vic HA clade definitions. Table S4: A(H1N1)pdm09 NA clade definitions. Table S5: A(H3N2) NA clade definitions. Table S6: B/Vic NA clade definitions. [file IRV-20-e70230-s001.pdf]

Supplementary Materials

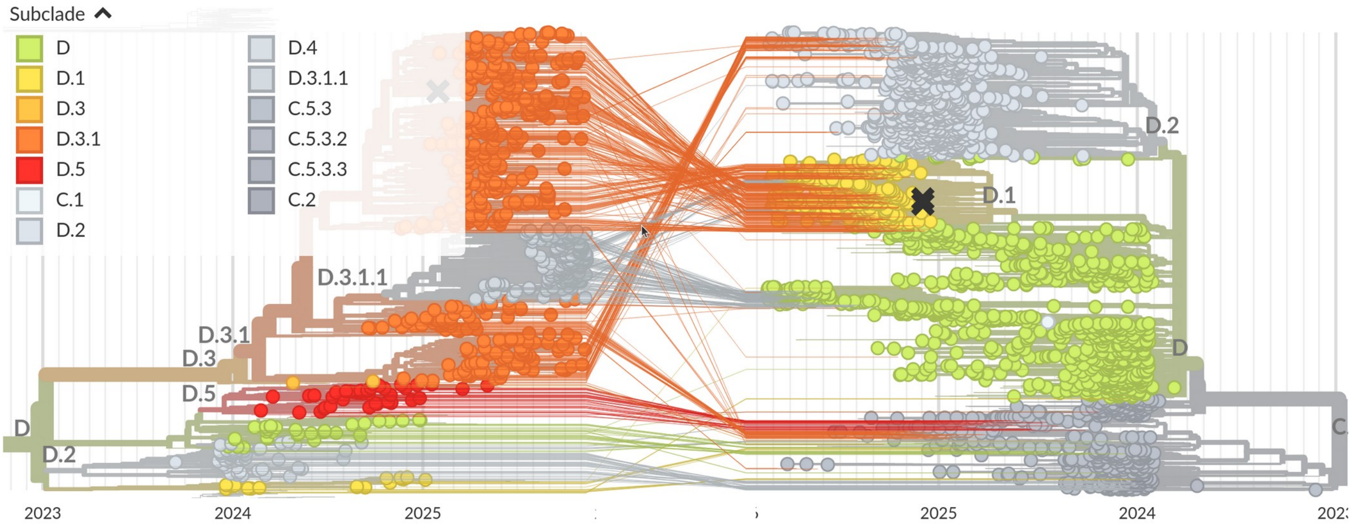

Supplementary figure 1: Tangle-gram of recent H1N1pdm isolates focusing on subclade H1-D of HA on the left with the NA tree on the right. Subclade nomenclatures for both segments allows for straightforward identification of mulit-segment constellations.

Tables

TABLE S1 A(H3N2) HA clade definitions

Subclade	Clade	full subclade name
A	3C	A
A.2	3C.2	A.2
A.3	3C.3	A.3
A.3.2	3C.3b	A.3.2
B	3C.2a	A.2.1
B.1	3C.2a1	A.2.1.1
B.1.1	3C.2a1a	A.2.1.1.1
B.1.2	3C.2a1b	A.2.1.1.2
B.1.2.1	3C.2a1b.1	A.2.1.1.2.1
B.1.2.1.1	3C.2a1b.1a	A.2.1.1.2.1.1
B.2	3C.2a2	A.2.1.2
B.3	3C.2a3	A.2.1.3
B.4	3C.2a4	A.2.1.4
C	3C.3a	A.3.1
C.1	3C.3a1	A.3.1.1
D	3C.2a1b.1b	A.2.1.1.2.1.2
E	3C.2a1b.2	A.2.1.1.2.2
E.1	3C.2a1b.2a	A.2.1.1.2.2.1
E.2	3C.2a1b.2b	A.2.1.1.2.2.2
F	1	A.2.1.1.2.2.1.1
F.1	1a	A.2.1.1.2.2.1.1.1
F.1.1	1a.1	A.2.1.1.2.2.1.1.1.1
G	2	A.2.1.1.2.2.1.2
G.1	2a	A.2.1.1.2.2.1.2.1
G.1.1	2a.1	A.2.1.1.2.2.1.2.1.1
G.1.1.1	2a.1a	A.2.1.1.2.2.1.2.1.1.1

G.1.1.2	2a.1b	A.2.1.1.2.2.1.2.1.1.2
G.1.2	2a.2	A.2.1.1.2.2.1.2.1.2
G.1.3	2a.3	A.2.1.1.2.2.1.2.1.3
G.1.3.1	2a.3a	A.2.1.1.2.2.1.2.1.3.1
G.1.3.2	2a.3b	A.2.1.1.2.2.1.2.1.3.2
G.2	2b	A.2.1.1.2.2.1.2.2
G.2.1	none	A.2.1.1.2.2.1.2.2.1
G.2.2	none	A.2.1.1.2.2.1.2.2.2
G.3	2c	A.2.1.1.2.2.1.2.3
G.4	2d	A.2.1.1.2.2.1.2.4
J	2a.3a.1	A.2.1.1.2.2.1.2.1.3.1.1
J.1	none	A.2.1.1.2.2.1.2.1.3.1.1.1
J.1.1	none	A.2.1.1.2.2.1.2.1.3.1.1.1.1
J.2	none	A.2.1.1.2.2.1.2.1.3.1.1.2
J.2.1	none	A.2.1.1.2.2.1.2.1.3.1.1.2.1
J.2.2	none	A.2.1.1.2.2.1.2.1.3.1.1.2.2
J.2.3	none	A.2.1.1.2.2.1.2.1.3.1.1.2.3
J.2.4	none	A.2.1.1.2.2.1.2.1.3.1.1.2.4
K	none	A.2.1.1.2.2.1.2.1.3.1.1.2.4.1
J.2.5	none	A.2.1.1.2.2.1.2.1.3.1.1.2.5
J.3	none	A.2.1.1.2.2.1.2.1.3.1.1.3
J.4	none	A.2.1.1.2.2.1.2.1.3.1.1.4

TABLE S2 A(H1N1)pdm09 HA clade definitions

Subclade	Clade	full subclade name
A	6B.1A.5a	A
B	5a.1	A.1
C	5a.2	A.2
C.1	5a.2a	A.2.1
C.1.1	5a.2a.1	A.2.1.1
C.1.2	none	A.2.1.2
C.1.3	none	A.2.1.3
C.1.4	none	A.2.1.4
C.1.5	none	A.2.1.5
C.1.6	none	A.2.1.6
C.1.7	none	A.2.1.7
C.1.7.1	none	A.2.1.7.1
C.1.7.2	none	A.2.1.7.2
C.1.8	none	A.2.1.8
C.1.9	none	A.2.1.9
C.1.9.1	none	A.2.1.9.1
C.1.9.2	none	A.2.1.9.2
C.1.9.3	none	A.2.1.9.3
C.1.9.4	none	A.2.1.9.4
D	none	A.2.1.1.1
D.1	none	A.2.1.1.1.1
D.2	none	A.2.1.1.1.2
D.3	none	A.2.1.1.1.3
D.3.1	none	A.2.1.1.1.3.1
D.3.1.1	none	A.2.1.1.1.3.1.1
D.4	none	A.2.1.1.1.4
D.5	none	A.2.1.1.1.5

TABLE S3 B/Vic HA clade definitions

Subclade	Clade	full subclade name
A	V1A	A
A.1	V1A.1	A.1
A.2	V1A.2	A.2
A.3	V1A.3	A.3
A.3.1	V1A.3a	A.3.1
A.3.1.1	V1A.3a.1	A.3.1.1
A.3.2	none	A.3.2
A.3.3	none	A.3.3
B	V1B	B
C	V1A.3a.2	A.3.1.2
C.1	none	A.3.1.2.1
C.2	none	A.3.1.2.2
C.3	none	A.3.1.2.3
C.3.1	none	A.3.1.2.3.1
C.3.2	none	A.3.1.2.3.2
C.4	none	A.3.1.2.4
C.5	none	A.3.1.2.5
C.5.1	none	A.3.1.2.5.1
C.5.2	none	A.3.1.2.5.2
C.5.3	none	A.3.1.2.5.3
C.5.4	none	A.3.1.2.5.4
C.5.5	none	A.3.1.2.5.5
C.5.6	none	A.3.1.2.5.6
C.5.6.1	none	A.3.1.2.5.6.1
C.5.7	none	A.3.1.2.5.7

TABLE S4 A(H1N1)pdm09 NA clade definitions

Subclade	full subclade name
A	A
A.1	A.1
A.1.1	A.1.1
B	A.1.1.1
B.1	A.1.1.1.1
B.2	A.1.1.1.2
B.2.1	A.1.1.1.2.1
B.3	A.1.1.1.3
B.3.1	A.1.1.1.3.1
B.3.1.1	A.1.1.1.3.1.1
B.3.1.2	A.1.1.1.3.1.2
C	A.1.1.1.3.2
C.1	A.1.1.1.3.2.1
C.2	A.1.1.1.3.2.2
C.3	A.1.1.1.3.2.3
C.4	A.1.1.1.3.2.4
C.5	A.1.1.1.3.2.5
C.5.1	A.1.1.1.3.2.5.1
C.5.1.1	A.1.1.1.3.2.5.1.1
C.5.2	A.1.1.1.3.2.5.2
C.5.3	A.1.1.1.3.2.5.3
C.5.3.2	A.1.1.1.3.2.5.3.2
C.5.3.3	A.1.1.1.3.2.5.3.3
D	A.1.1.1.3.2.5.3.1
D.1	A.1.1.1.3.2.5.3.1.1
D.2	A.1.1.1.3.2.5.3.1.2

TABLE S5 A(H3N2) NA clade definitions

Subclade	full subclade name
A	A
A.1	A.1
A.2	A.2
A.2.1	A.2.1
A.2.2	A.2.2
A.2.2.1	A.2.2.1
A.2.2.2	A.2.2.2
B	A.2.2.3
B.1	A.2.2.3.1
B.1.1	A.2.2.3.1.1
B.2	A.2.2.3.2
B.2.2	A.2.2.3.2.2
B.3	A.2.2.3.3
B.4	A.2.2.3.4
B.4.1	A.2.2.3.4.1
B.4.2	A.2.2.3.4.2
B.4.2.1	A.2.2.3.4.2.1
B.4.2.2	A.2.2.3.4.2.2
B.4.2.3	A.2.2.3.4.2.3
B.4.3	A.2.2.3.4.3
B.4.4	A.2.2.3.4.4

TABLE S6 B/Vic NA clade definitions

Subclade	full subclade name
A	A
A.1	A.1
A.1.1	A.1.1
A.1.1.1	A.1.1.1
A.1.1.2	A.1.1.2
A.1.1.3	A.1.1.3
A.1.1.4	A.1.1.4
B	A.1.2
B.1	A.1.2.1
B.2	A.1.2.2
B.3	A.1.2.3
B.4	A.1.2.4
B.5	A.1.2.5
B.6	A.1.2.6
B.7	A.1.2.7
B.7.1	A.1.2.7.1
B.7.2	A.1.2.7.2
B.7.3	A.1.2.7.3
B.8	A.1.2.8
